# Mechanisms of human cerebellar dysmetria: experimental evidence and current conceptual bases

**DOI:** 10.1186/1743-0003-6-10

**Published:** 2009-04-13

**Authors:** Mario Manto

**Affiliations:** 1 Laboratoire de Neurologie Expérimentale, FNRS-ULB, Bruxelles, Belgium

## Abstract

The human cerebellum contains more neurons than any other region in the brain and is a major actor in motor control. Cerebellar circuitry is unique by its stereotyped architecture and its modular organization. Understanding the motor codes underlying the organization of limb movement and the rules of signal processing applied by the cerebellar circuits remains a major challenge for the forthcoming decades. One of the cardinal deficits observed in cerebellar patients is dysmetria, designating the inability to perform accurate movements. Patients overshoot (hypermetria) or undershoot (hypometria) the aimed target during voluntary goal-directed tasks. The mechanisms of cerebellar dysmetria are reviewed, with an emphasis on the roles of cerebellar pathways in controlling fundamental aspects of movement control such as anticipation, timing of motor commands, sensorimotor synchronization, maintenance of sensorimotor associations and tuning of the magnitudes of muscle activities. An overview of recent advances in our understanding of the contribution of cerebellar circuitry in the elaboration and shaping of motor commands is provided, with a discussion on the relevant anatomy, the results of the neurophysiological studies, and the computational models which have been proposed to approach cerebellar function.

## 

Optimal strategies are required to perform motion with accuracy, given the highly complex non-linear biomechanical features of the human body, including the muscles and joints, and the numerous interactions with the environment. The central nervous system (CNS) copes with noise and delays, which are inherent to biology and also motion. The notion of noise in biological signals includes both the input noise and the internal noise [1,2]. Noise may also fluctuate with time or according to a particular sensori-motor context. Therefore, a high degree of adaptability and modifiability in the operational mechanisms underlying motor control is required, especially for learning procedures.

The cerebellum plays fundamental roles in action control and motor learning [3]. Cerebellar circuitry controls movement rate, smoothness, and coordination aspects [4]. Several theories have been proposed these last 4 decades, emerging mainly from the bioengineering field. These computational theories take into account the division of cerebellum in microcircuits and the connectivity of the different cerebellar regions with the motor/prefrontal cerebral cortex, the thalamus, the brainstem and the spinal cord [5,6].

This review will focus on motor dysmetria of limbs, a cardinal sign of cerebellar diseases. I examine the current conceptual bases and the experimental findings. This review does not analyze the literature of ocular reflexes/oculomotor control and does not consider the mechanisms of gait ataxia. The neuropsychological deficits observed in cerebellar patients ("cerebellar cognitive affective syndrome", dysmetria of thought) have been reviewed recently elsewhere [see [7]].

### Definition of dysmetria

Dysmetria designates the lack of accuracy in voluntary movements [8]. The most common form of errors in metrics of motion is *hypermetria*, defined as the overshoot of an aimed target during voluntary movement (Figure 1). Cerebellar patients can also exhibit an undershoot or premature arrest before the target, called *hypometria*. In some patients, both forms of dysmetria are present and in others hypermetria may be followed by hypometria during an aberrant recovery following an acute cerebellar lesion such as a cerebellar stroke. Initiation of movement is often delayed in cerebellar disorders [9,10]. This is common in patients exhibiting severe dysmetria associated with degenerative disorders of the cerebellum. Cerebellar dysmetria occurs proximally and distally in upper and lower limbs, affects both single-joint and multi-joint movements and is larger for movements performed as fast as possible (Figure 2). Trajectories of cerebellar patients are characterized by an increased curvature [11,12]. Trajectories of the wrist during multi-joint reaching movements are abnormally curved, with tendencies to move a joint at a time [13]. Dysmetria is often followed by corrective movements. Unlike kinetic tremor, the second cardinal sign of a cerebellar disease, hypermetria worsens when the mass of the limb is increased. In cerebellar hypermetria, kinematic profiles of single-joint movements are often asymmetrical, meaning that the deceleration peak is higher than the acceleration peak, resulting in acceleration/deceleration ratios lower than 1 (Figure 3). In addition, acceleration time or deceleration time may also be prolonged [10,14]. Moreover, dysmetria is often associated with impaired rhythm generation and increased variability in movement. Dysmetric movements show an increased variability very early in the movement trajectory, which is not influenced by visual feedback [15]. However, the large errors near the aimed target are increased in darkness. Despite the fact that patients improve their performance under visual guidance, the visual correction mechanism per se is abnormal, with the end phase of the movement prolonged and excessive deviations or directional changes in the path [15]. Although hypermetric movements are very suggestive of a cerebellar deficit, they are not completely specific. They can be encountered in case of thalamic lesion, for instance.

**Figure 1:**
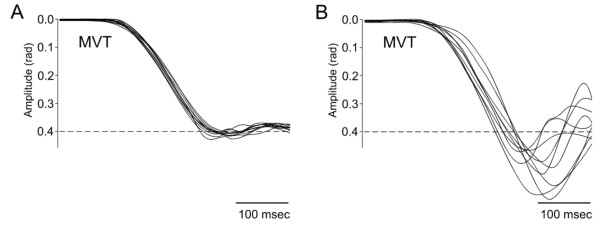
**Cerebellar hypermetria**. Superimposition of 9 fast wrist flexion movements in a control subject [A] and a cerebellar patient [B]. Movements (MVT) are accurate in A and are hypermetric in B (overshoot of the target). Aimed target (dotted lines) located at 0.4 rad from the start position corresponding to a neutral position of the joint. The target is visually displayed.

**Figure 2:**
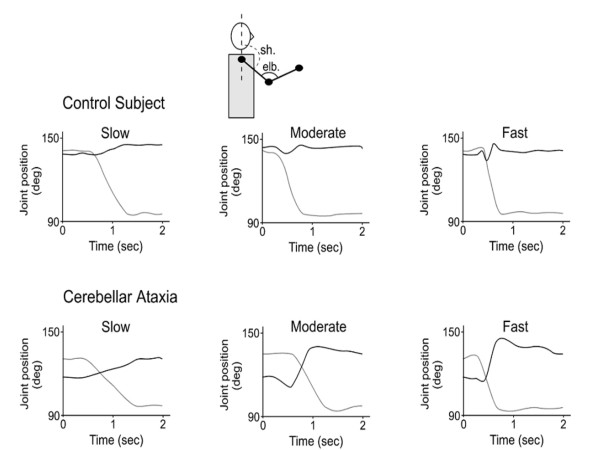
**Effects of increasing velocities on kinematics of the upper limb pointing movements in a control subject (upper panels) and a cerebellar patient (lower panels)**. Subjects are seated and comfortably restrained in order to allow only shoulder and elbow movements. They are asked to perform a vertical pointing movement towards a fixed target at various speeds. The target is located in front of the subjects at a distance of 85% of total arm length. In the patient, deficits in angular motion are enhanced with increasing velocities, especially the increased angular motion of elbow resulting in overshoot (hyperextension of the elbow). Black lines: angular position of the elbow; grey lines: angular position of the shoulder. Abbreviations: sh: shoulder angle, elb: elbow angle.

**Figure 3:**
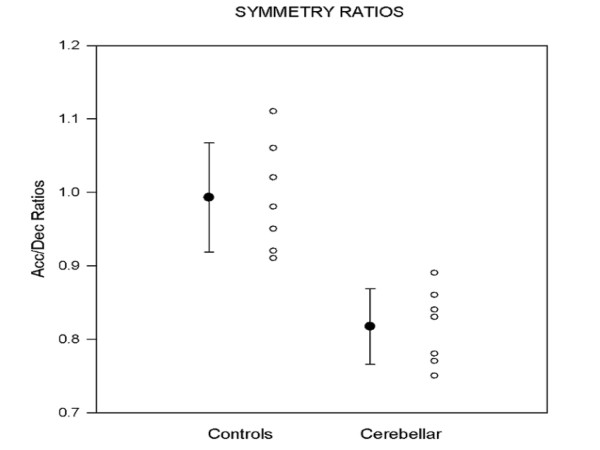
**Asymmetry in kinematics of fast wrist flexion movements in cerebellar patients exhibiting hypermetria**. Values correspond to ratios of Acceleration Peaks divided by Deceleration Peaks. Mean +/- SD and individual ratios are shown. Data from n = 7 ataxic patients; mean age: 53.2 +/- 5.7 years. Control group: n = 7 subjects; mean age: 54.5 +/- 6.1 years. Aimed target: 15 degrees; n = 10 movements per subject.

### The anatomy and physiology of the cerebellum

The cerebellum is composed of a mantle of grey zone, surrounding white matter in which cerebellar nuclei are embedded. Cerebellum is divided in 10 lobules (I-X). Each region of the cerebellum has thus a unique connectivity, despite the apparent homogeneous cytoarchitecture [16]. Three main types of fibers enter in the cerebellum: the climbing fibers, the mossy fibers and the diffusely distributed cholinergic/monoaminergic afferents (Figure 4). Noteworthy, the inferior olive is the single source of climbing fiber inputs to the cerebellum, and houses cells with oscillatory properties [17]. By contrast, mossy fibers arise from a large spectrum of ipsilateral and contralateral sources.

**Figure 4:**
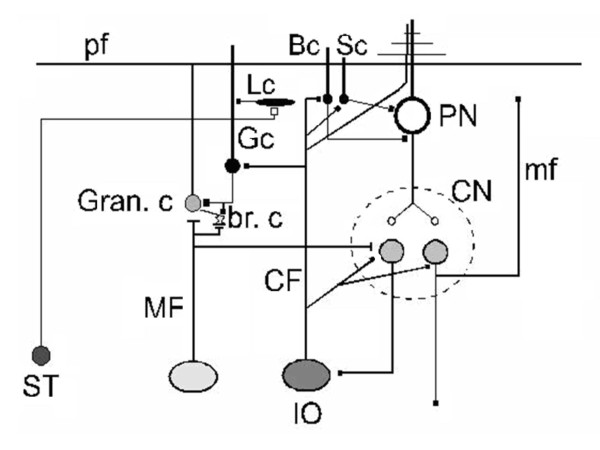
**Wiring diagram of the cerebellar circuitry**. Purkinje neurons are the sole output of the cerebellar cortex. Basket cells supply the inhibitory synapses via a synapse called "pinceau", stellate cells supply the inhibition to Purkinje cell dendrites. Lugaro cells are activated by serotoninergic fibers and inhibit Golgi cells. In addition to the illustrated serotoninergic afferences, cerebellar cortex receives other aminergic inputs (acetylcholine, dopamine, norepinephrine, histamine) or peptidergic projections (peptides such as neurotensin). These fibers project sparsely throughout the granular and molecular layers to contact directly the Purkinje neurons and other cerebellar neurons. Abbreviations: ST: serotoninergic fiber, pf: parallel fiber, Gran. c: granule cell, MF: mossy fiber, br. c: unipolar brush cell, CF: climbing fiber, IO: inferior olive, Gc: Golgi cell, Lc: Lugaro cell, Bc: basket cell, Sc: stellate cell, PN: Purkinje neuron; CN: cerebellar nucleus, mf: recurrent mossy fiber from nuclear cell.

#### Cerebellar cortex and microcomplexes

Cerebellar cortex is characterized by a laminated geometrical structure. The Purkinje cells represent the unique output of cerebellar cortex, targeting nuclear neurons [18]. The excitation of Purkinje neurons is balanced by the activity of inhibitory interneurons located in the molecular (basket cells, stellate cells) and granular layers of the cortex (Golgi cells and Lugaro cells). In human, the number of Purkinje cells has been estimated to about 15 millions [19]. The axon of a Purkinje neuron gives off about 500 terminals which contact 30–40 nuclear cells. Each nuclear cell receives projections from 800–900 Purkinje neurons.

Granule cells are the most numerous neurons in the human brain, the population being estimated to about 10^10^–10^11 ^cells [19,20]. These neurons have four to five dendrites and make synapses with the enlarged excitatory terminals of mossy fibers ("rosettes"). Each granule neuron receives mossy terminals via only four to five excitatory synapses, suggesting *a sparse coding (small convergence number)*. This code can be defined as a neural code in which the fraction of active neurons is low at a given time. Granule cells have low levels of spontaneous activities. A single impulse in a mossy fiber tends to induce burst spikes in a granule cell [21,22]. However, granule cells are usually active only briefly following a sensory stimulus. Sparse coding could reduce interference issues between tasks being learned by a subject [16]. Sparse coding could also enhance storage capacity [16,21]. This is based on the well know divergence of mossy fiber input to the granule layer and the minimal redundancies between granule cell discharges [22]. To maintain the low mean firing rate compatible with a sparse code, an activity-dependent homeostatic mechanism would set the cells' thresholds [22]. Each granule cell has a thin axon ascending in the molecular layer and which divides in 2 opposites branches called parallel fibers, running along the folia. The length of a parallel fiber has been estimated to 4–6 mm [23]. Local excitation of a parallel fiber bundle stimulates Purkinje cells over a distance of more than 3 mm. A single parallel fiber passes through the dendrites of more than 400 Purkinje cells, making contacts with the dendritic spines of at least 300 Purkinje neurons [24]. Dendrites of Purkinje neurons are disposed within planes perpendicular to the long axis of the folia. Each dendritic arborization of Purkinje neuron enters in contact with more than 100.000 parallel fibers. Parallel fiber beams can bridge and make functional links between cerebellar nuclei (Figure 5) [25], with a beam exciting the dendrites of Purkinje, basket, stellate and Golgi cells. Basket and stellate axons run tangentially to either side of the transverse parallel fiber beam, inhibiting Purkinje cells in the 'flanks' of the beam [26]. Links across the interpositus and dentate nuclei would effectively connect reach, grasp and reflex sensitivity. This is based on the fact that each nucleus has a separate somatotopical representation of the body. Head is caudal, tail rostral, trunk lateral and extremities medial [27-29]. In each nucleus, distal and proximal muscles are represented and these regions can be coordinated by beams of parallel fibers linking Purkinje cells belonging to distinct functional units oriented along planes perpendicular to the longitudinal axis of the folia. This organization is the anatomical substratum allowing the coordination of wrist, elbow and shoulder joint during motion. Indeed, the length of parallel fibers is sufficient to ensure the connection of Purkinje cells projecting to different nuclei, permitting the coordination of the corresponding functions such as control of locomotion, modulation of reflex activity and reaching-grasping.

**Figure 5:**
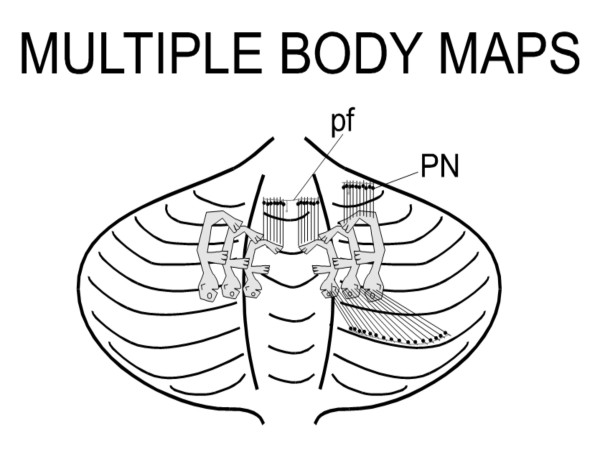
**Multiple body maps in the cerebellum**. Each cerebellar nucleus has a complete map of the body, with head located posteriorly, limbs medially and trunk laterally. Thanks to the parallel fibers (pf, issued from granule cells) linking together Purkinje neurons (PN) projecting to distinct body areas, myotomes can be interconnected during motor tasks. Parallel fibers are long enough to link together Purkinje neurons projecting to different portions within one nuclear body map, and multiple maps. The contacts between parallel fibers and the dendrites of cortical inhibitory interneurons are not illustrated. Adapted from Thach, 2007.

The inferior olive transmits signals to a well-defined cluster of sagittally organized Purkinje cells, which project to given areas in nuclei. These latter send a feedback projection to the inferior olive (nucleo-olivary projections). Seven parallel longitudinal zones are organized on each side of the cerebellum (A, B, C1, C2, C3, D1, D2). The parasagittally striped organization of the cerebellum is also found for the expression of acetylcholinesterase and other molecules such as zebrin II [see [30]]. The C3 zone receives inputs from the receptive fields in forelimb skin and contains 30–40 longitudinal *microzones*, each 50 to 150 μm wide [16]. These microzones are the functional units of the cerebellar cortex. *Microcomplexes *refer to the combination of a microzone and the related structures: small groups of neurons in a cerebellar or vestibular nucleus, the inferior olive and neurons in red nucleus [16]. The human cerebellum might contain about 5000 microcomplexes. Climbing fibers in nearby microzones are activated from neighbouring skin areas, making a somatotopic map of the ipsilateral forelimb skin [16]. The loop is closed in a way, since microzones project to adjacent cell groups in the anterior interpositus nucleus which controls movements having a close relationship with the climbing fibers' receptive fields.

#### Cerebellar nuclei

They represent the sole output from cerebellar circuits, bringing signals in particular to brainstem nuclei, thalamic nuclei, motor cortex, premotor cortex and prefrontal association cortex via the cerebellothalamocortical tracts (Figure 6, Figure 7). Cerebellar nuclei project back to the overlying cerebellar cortex, with a mediolateral and rostrocaudal pattern of nucleocortical projections reflecting the corticonuclear projections [31].

**Figure 6:**
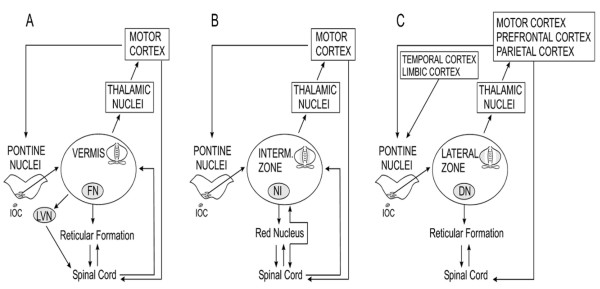
**Comparison of anatomical connections of the vermal zone (A), the intermediate zone (B) and the lateral zone of the cerebellum (C)**. The midline zone and the intermediate zone receive direct informations from the spinal cord, unlike the lateral cerebellum. Abbreviations: IOC: inferior olivary complex, LVN: lateral vestibular nucleus, FN: fastigial nucleus, NI: nucleus interpositus, DN: dentate nucleus.

**Figure 7:**
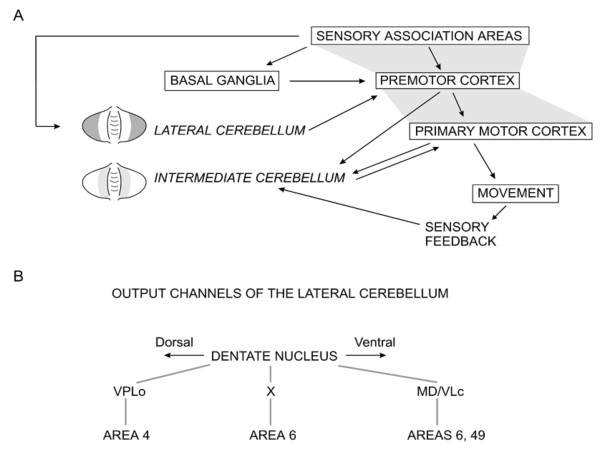
**A: According to the model of Allen and Tsukahara (1974), the intermediate zone of the cerebellar hemisphere contributes to movement execution by monitoring actual sensory feedback and processing error signals that compensate for prediction errors in movement planning**. The lateral zone of the cerebellar hemisphere participates in the planning and programming of movements by integrating sensory information. B: Output channels in the dentate nucleus. Distinct areas of the dentate nucleus project predominantly upon different regions of the contralateral cerebral cortex, via thalamic nuclei (MD/VLc: medial dorsal/ventralis lateral pars caudalis nuclei, 'area X', VPLo: nucleus ventralis posterior lateralis pars oralis). Dorsal portions of the dentate nucleus project mainly upon area 4.

In primates, fastigial nuclei project -*although not exclusively*- on both sides to the hindlimb area of the motor cortex and the parietal cortex [32]. Interpositus nuclei are connected with the trunk areas of the motor cortex/premotor cortex [32]. Dentate nuclei have contralateral projections to the forelimb zones of the motor cortex/premotor cortex/prefrontal association cortex [32]. Ventral areas of the dentate nuclei tend to project upon the prefrontal cortex, in particular zone 9 and 46 which are involved in working memory and guidance of behaviour based on transiently stored information, while dorsal areas send projections primarily to M1 area (Figure 7) [33]. Functionally, fastigial nuclei are especially concerned with eye movements, as well as upright stance and gait; the interpositus nuclei play key-roles in the modulation of reflexes, such as stretch, contact and placing reflexes; dentate nuclei are mainly involved in voluntary movements of the extremities such as single-joint and multi-joint goal-directed movements towards a fixed or moving target [25].

#### Patterns of neuronal discharges in cerebellar circuits

Olivary cells fire between 1 and 10 Hz, with a mean frequency close to 1 Hz in most species [34]. The upper frequency is limited by the long after-hyperpolarization which lasts about 100 msec. Simple spikes of Purkinje cells could determine the activity of the cerebellar nuclei, and therefore govern cerebellar outflow. Simple spike activity is mainly driven by the mossy fiber inputs to granule cells. Its modulation is low during passive movements and high during active movements [35,36].

The complex spikes would serve *as error signals *to adjust the simple spike discharges if an error occurs [37]. Simultaneous electrical stimulation of mossy and climbing fibers depresses the parallel fiber-Purkinje cell synapses which are concurrently active (the so-called *long-term depression *LTD, a form of synaptic plasticity [37]. LTD is associated with a decrease of the post-synaptic sensitivity to glutamate caused by removal of AMPA receptors by endocytosis [38]. LTD plays an essential role in the cerebellum's error-driven learning mechanism [16]. In order to have a stable memory process, an opposing process must balance LTD: long-term potentiation (LTP). Post-synaptic LTP is able to reset post-synaptic LTD [39]. Predominance of silent granule synapses is in agreement with a key-role of LTP for new learning [1]. For numerous tasks, learning must initially proceed via LTP in either the direct or indirect pathway from granule cells to Purkinje neurons. The first pathway would increase the excitability of the Purkinje cell, by contrast with the second pathway.

Despite the inhibitory role exerted by Purkinje neurons upon cerebellar nuclei, the neurons in these latter fire spontaneously between 10 and 50 Hz. In absence of motion, high rates of discharges of about 40–50 Hz are common [25]. During motion, firing rates increase and decrease above and below the baseline. This contributes to the modulation of the sensitivity of given targets according to a specific sensorimotor context.

Recordings in the fastigial nuclei indicate that they can be divided into a rostral and a caudal zone [40]. The rostral zone is in charge of the descending control of somatic musculature, controls head orientation and combined eye-head gaze shifts. The caudal zone controls oculomotor functions (saccades, smooth pursuit) [41]. There are direct and indirect evidence that discharges in the interpositus nucleus are related to the antagonist muscle being used [25,42-44]. Interpositus neurons modulate their activities in relation to sensory feedback including that from oscillations in movements [45-47]. Interpositus nucleus might select the degree of reciprocal versus co-contraction pattern in a given task [43]. Moreover, the interpositus nuclei regulate the discharge of gamma motor neurons [48] and the excitability of the anterior horn in the spinal cord [49]. The temporary inactivation of interpositus nucleus using a cooling procedure induces tremor which is sensitive to proprioceptive feedback but insensitive to vision [45]. The cooling induces a 3–5 Hz action tremor as the animals attempt to reach and grasp food, supporting the idea that the interpositus nucleus uses abundant afferent inputs to generate *predictive signals*. Monzée and colleagues have shown in monkey that injections of muscimol in the region corresponding to the anterior interpositus nucleus induce a pronounced tremor and dysmetria of the ipsilateral arm when the animal performs unrestrained reaching and grasping movements [50]. Cells with anticipatory and reflex-like responses in a lift and hold task are located in the dorsal anterior interpositus and not in the dentate nucleus [51]. Hore and Flament (1986) have observed a terminal tremor during targeted limb movements after cooling of cerebellar nuclei [52]. They have hypothesized that cerebellum stabilizes limbs during a maintained posture or after a brisk movement. To counteract oscillations that would otherwise contaminate the termination of movement, the CNS generates bursts of muscle activity which anticipate the oscillations. Cooling of cerebellar nuclei interferes with the normal *predictive *nature of these suppressive bursts [53]. In absence of adequately timed suppressive bursts, the position of the limb is driven by non-anticipatory and transcortical stretch responses [54]. Transcortical reflex activities may even reinforce oscillations, instead of damping them. Repetitive TMS of the primary motor cortex induces a cerebellar-like tremor which is attributed to the deficiency in the generation of predictive responses [55].

Single-unit studies have demonstrated that the neuronal activity in the dentate nucleus precedes the onset of movement and may also start before the discharges in the contralateral motor cortex [56]. In particular, dentate neurons are active preferentially when motion is triggered by a mental association with visual or auditory stimuli [25]. A key-experiment was performed by Thach in 1978. The author recorded the activities in the motor cortex, the dentate nucleus, the interpositus nucleus and limb muscles in monkeys [56]. When an external force disturbed wrist position, the order of firing was: muscles, interpositus, motor cortex, dentate. When motion was triggered by light, the order of activity was: dentate, motor cortex, interpositus, muscles. These data strongly suggest that the interpositus is involved in corrective movements initiated by the feedback of the movement itself, whereas the dentate nucleus contributes to the initiation of a movement which is triggered by stimuli mentally associated with the task. Anterior lesions might impair more specifically grasping, and posterior lesions could generate especially reaching deficits [57]. Inactivation of the dentate nuclei result in *delayed reaction times *in movements triggered by light or sound [58], similarly to what is observed in cerebellar patients.

Cerebellar input exerts a facilitatory drive upon the contralateral cerebral cortex. Experimentally, cerebellar lesions depress the excitability of the contralateral motor cortex, both in human and in rodents (Figure 8) [59,60]. Non-invasive transcranial activation of neural structures using electrical and magnetic stimulation (TMS: transcranial magnetic stimulation) has allowed the investigation of the cerebello-thalamo-cortical pathway in humans. Ugawa et al. have demonstrated significant gain of EMG responses at an inter-stimulus interval (ISI) of 3 ms (facilitatory effect) [61]. Conditioning magnetic stimulus of the cerebellum suppresses motor cortex excitability 5–8 msec later. This method activates the unilateral cerebellar structures under the coil. Impaired facilitation and enhanced inhibition within motor cortex have been observed repeatedly in patients presenting cerebellar lesions [62-66]. Hemicerebellectomy is associated with higher motor thresholds contralateral to the cerebellar lesion. The cerebellum influences also the excitability of sensitive areas in the brain. Indeed, it has been demonstrated that the N24 and later components in somatosensory evoked potentials are markedly reduced in case of absence of cerebellar input, suggesting that the cerebellar circuits influence directly the excitability of the parietal cortex [67].

**Figure 8:**
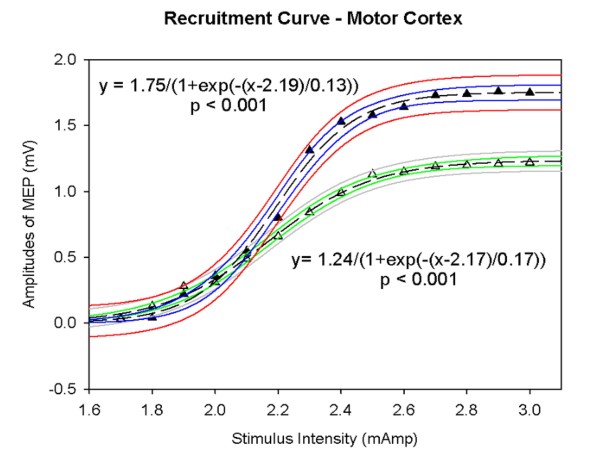
**Decreased excitability of the motor cortex contralaterally to the ablation of the left hemicerebellum in a rat, as revealed by the study of recruitment curves of corticomotor responses in the gastrocnemius muscle**. Recordings in the gastrocnemius muscle following incremental electrical stimulation of the motor cortex. Plots correspond to the amplitude of motor evoked potentials as a function of stimulus intensity. Filled triangles: stimulation of left motor cortex, open triangles: stimulation of right motor cortex. Fitting with a sigmoidal curve (3 parameters). 95% prediction band and 95% confidence band are illustrated. Amplitudes of recorded motor evoked potentials (MEPs) are expressed in mV.

We recently found that trains of transcranial direct current stimulation (tDCS) applied over the motor cortex, a technique which is known to facilitate the overall neural activity of the stimulated area [68,69], can revert the decrease of excitability induced by an extensive and acute unilateral cerebellar lesion [70]. tDCS probably restores the balance between excitatory and inhibitory circuits in case of hemicerebellar ablation. This opens the possibility of treating human cerebellar dysmetria with tDCS.

### Computational models

The main theories of cerebellar function and their respective assumptions are summarized in table 1[25,71-77]. The works of Marr and Albus have exerted a strong influence on computational models of cerebellar function these last decades [16]. Another attractive model is based upon the adaptative filter hypothesis. The adaptative filter, developed by Fujita [71] following the Marr-Albus framework, is a signal-processing device transforming a set of temporally varying signals into another [1]. Inputs to the filter are split into components weighted individually and then recombined to generate the filter's output. These weights determine the output. This is a central task for the adaptative filter [1]. This is done by a teaching signal and a learning rule for changing weight values. In case of the cerebellar circuitry, if the firing of parallel fibers is positively correlated with the firing of climbing fibers, the weight is reduced (LTD). The reverse leads to an increase in the weight (LTP). No change occurs if the firings are uncorrelated. This corresponds to the covariance learning rule [78]. This rule does not distinguish LTP from LTD, considering that both are part of the same computational process.

**Table 1:** Theories of cerebellar functions

Theory	Assumptions	Selected referenceq
Adaptative filter hypothesis	Based upon Marr-Albus theory. Transformation of sets of signals into others. Components are weighted individually and then recombined to minimise the errors in performance caused by unavoidable noise.	Fujita, 1982 [71]

Internal models	The cerebellum contains neural representations to emulate movement. Internal models reproduce the dynamic properties of body parts.	Wolpert et al., 1998 [72]
*Forward model*	The model predicts the next state given the current state and the motor command.	
*Inverse model*	The model inverts the system by providing the motor command that will cause the desired change in state.	

Tonic reinforcer	The cerebellum tunes the intensities of agonist/antagonist/synergist muscles. Cerebellum exerts an excitatory influence upon extra-cerebellar targets.	Eccles et al., 1967 [73] Bastian and Thach, 2002 [25]

Cerebellar timer	Cerebellum is the main site of temporal representation of action.	Braitenberg, 1967 [74] Ivry and Spencer, 2004 [75]

Wave-variable processor	The cerebellum contributes to a servo-motor mechanism.	Massaquoi and Slotine, 1996 [76]

Sensory processor	The cerebellum monitors and adjusts the acquisition of sensory information.	Bower, 1997 [77]

The adaptative-filter model has 2 main differences with the Marr-Albus theory, making this a suitable candidate for modelling cerebellar microcircuits. First, the signal-processing algorithm is used in many practical applications. In this sense, it is considered as a model whose functionality is demonstrated. It depends on the connectivity with other structures, which is very consistent with the anatomical organization of cerebellar circuits. Second, it involves time-varying signals and therefore addresses the key-issue of timing [1].

#### Internal models

It is widely accepted that expectations and estimates of future motor states are critical for performing fast coordinated movements. One of the main theories addresses a central issue in motor control, namely the intrinsic time delay of sensory feedback associated with motor commands and motion. Sensory-motor delays vary according to the modality and context, and may be in the order of 50–400 msec. Such delays imply that in-flight updating of motor commands using sensory feedback can never be ideal [4]. The cerebellum has therefore been proposed to contain neural representations or 'internal models' to emulate fundamental natural processes such as body movement [Figure 9] [3]. According to internal models, the motor cortex is able to perform an accurate movement using an internal feedback instead of the external feedback from the real control object [16]. The internal feedback is closely linked to the internal model of the object, built in the cerebellum in close cooperation with the cerebral cortex. This theory is supported by fMRI studies, TMS experiments and psychophysical studies. Indeed, the study of Kawato et al [79] using fMRI strengthens the hypothesis that the cerebellum implements a *forward model *for coordination and accuracy in motor tasks, employing a predictive information from one effector to ensure motor control of another one. Miall et al [80] have studied the effects of disrupting the cerebellum during a reach-to-target task using TMS. Stimuli were applied over the ipsilateral cerebellum during the reaction time of the subject who had to point to a previously observed target location following an auditory cue. Errors in the initial direction and the final position were consistent with the pointing movements being planned from an estimated hand position which was about 140 msec out of date. These data suggest that the cerebellum predictively updates a central state estimate. According to this hypothesis, clumsiness in cerebellar patients and dysmetria are due to *a malfunction in the predictive feedforward control *and/or to a disorder in the accurate appraisal of the consequences of motor commands. Internal models have the advantage to allow the brain to precisely control the movement without the need for sensory feedback [16].

**Figure 9:**
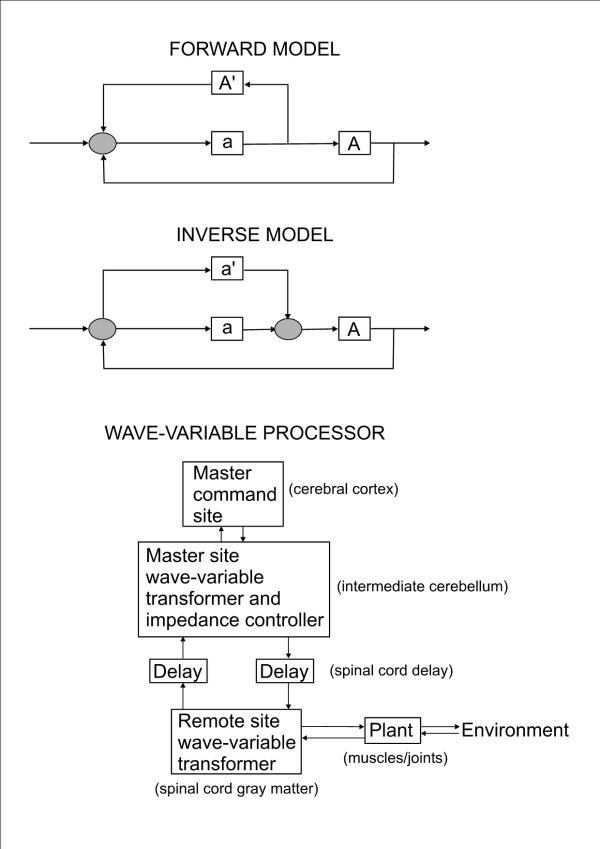
**Forward model-based control scheme (top panel) and inverse model-based control scheme (middle panel)**. Forward model: the message dedicated to the peripheral motor apparatus A is sent with an efference copy transmitted to the cerebellum A'. Instructions originating from higher motor centers (such as the premotor cortex) reach a comparator (grey circle). The comparator drives the motor cortex (a), which in turns drives lower motor centers in the brainstem and spinal cord. Efference copies are used to perform future predictions. Cerebellar microcircuits are necessary to learn how to make appropriately these predictive codes. Inverse model: A corresponds to the motor apparatus/control object. Cerebellar cortex working in parallel with the motor cortex and forming an internal model with a transfer function a' reciprocally equal to the dynamics of the control object (a' = 1/A). The input to the cerebellum is the desired trajectory, the output is the motor command. The bottom panel illustrates the model of the wave-variable processor for the intermediate cerebellum and the spinal cord gray matter. These structures contribute to motion control by processing control signals as wave variables. These wave variables are combinations of forward and return signals ensuring stable exchanges despite destabilizing signal transmission delays (adapted from [76].

#### Forward models

The cerebellum may function similarly to a 'forward model' by using *efference copies *of motor orders to predict sensory effects of movements. Accurate predictions would decrease the dependence on time-delayed sensory signals. Cerebellar circuitry would be necessary to learn to make appropriate predictions using error information about the discrepancies between the actual and predicted sensory consequences, not only for limb movements but also for postural adjustments [81,82]. Figure 10 shows a schematic view of the connections that could represent important elements of the model. The cortico-ponto-cerebellar tracts bring an efference copy of a motor command to the cerebellar cortex. The cerebellum would compute an expected sensory outcome, which would be sent to cerebral cortical areas via excitatory connections to the thalamus, and to the inferior olive via inhibitory connections. The inferior olive, which may receive a corollary discharge directly from the motor cortex, could operate as a sort of *comparator*, signalling errors to back to cerebellar cortex and training it to make correct predictions. Purkinje cell firings have several of the characteristics of a forward internal model of the arm. Indeed, Purkinje cell firing heralds the kinematics of motion. Purkinje cell discharges anticipate the kinematics of motion, in agreement with a prediction activity as demonstrated during circular manual tracking in monkey [83]. Experimental data suggest that Purkinje neurons from lobules IV-VI encode position, directional parameters and velocities of arm movements [83,84]. Purkinje cells might provide a prediction signal of the consequences of movement [85].

**Figure 10:**
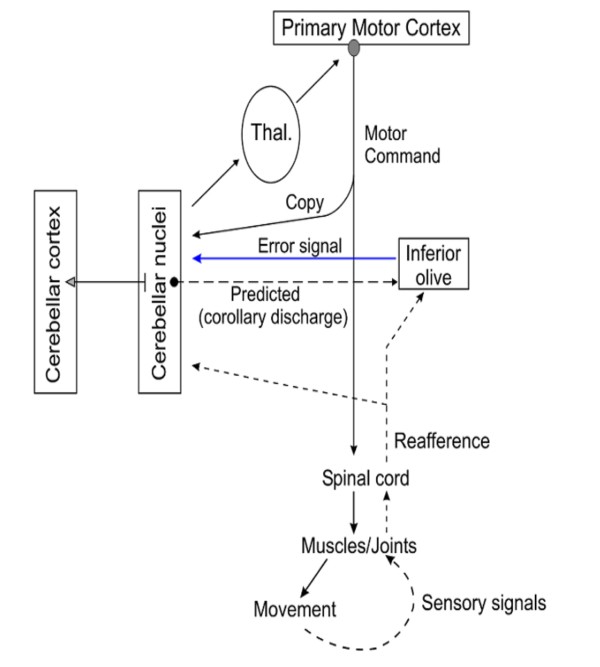
**Communication flows for information processing in forward models of motor coding**. Cerebellar modules receive an efference copy of motor commands via the corticopontocerebellar tract, in order to make predictions. Reafference signals and corollary discharges reach the comparator (inferior olive), which generates an error signal updating the plastic cerebellar microcircuits. Expected sensory outcomes are conveyed to the primary motor cortex via excitatory connections and to the inferior olive via inhibitory pathways.

Some of the most convincing evidence that the central nervous system (CNS) uses internal forward models in human motor behavior comes from studies dedicated to the control of grasping forces during manipulation of objects [86]. The rate of grip force development and the balance between the grip and load forces when grasping/lifting an object is programmed in order to meet the requirements due to physical object properties, such as weight, surface friction or shape. Cerebellar patients generate excessive grip forces in relation to loads and converging data suggest a distorted predictive force control in cerebellar disorders [86].

Experimental evidence suggesting the use of internal models for sensory signals has also been found in other species. In several teleosts, cerebellum-like structures predict the sensory consequences of the behaviour of the fish [87]. The suppression of self-generated electrosensory noise (reafference) and other predictable signals is performed partly by an *adaptive filter mechanism*, which could represent a more ubiquitous form of the modifiable efference copy mechanism.

#### Inverse models

According to this theory, the cerebellum would lodge an 'inverse model'. Here the input to the cerebellum would be the aimed trajectory, and the output would be a motor command. In order to train this type of model, error information would best be characterized in motor coordinates in 3 directions. In the laboratory, cerebellar patients exhibit difficulties in adapting to external force field, in agreement with the inverse dynamics hypothesis [88]. There are neurophysiological data supporting the existence of inverse models: Shidara and colleagues have shown that Purkinje cell activity during ocular movements are consistent with signals of an inverse model [89]. Although studies of the changes in Purkinje cell firings occurring when an external force load is changed from resistive to assistive during elbow movements are suggestive of inverse dynamics model, it should be noted that these experiments have not controlled limb kinematics or modified the magnitude of external loads [90]. To test the hypothesis that Purkinje cell firing is the output of an inverse dynamics model, forces must be changes while kinematics are kept constant. The study of Pasalar and colleagues [91] is consistent with the idea that Purkinje cells in cerebellar cortex code for kinematic (i.e. sensory state) but not dynamic information (i.e. muscle commands). The majority of Purkinje cells do not exhibit any modulation in the patterns of discharges as a function of force type or load. In addition, the directional tuning pattern seems unaffected, strengthening the idea of uncoupling between Purkinje cell firing and electromyographic (EMG) activity in limbs. One of the differences between cerebellar simple spike responses and those of motor cortical cells is the non uniform distribution of preferred directions across the workspace and the extensive overlap in the timing of the simple spike correlations with movement direction, distance and target position. These differences suggest that Purkinje cells handle kinematic information in a different way as compared to motor cortical neurons [84].

The intermediate cerebellum might learn internal models of body mechanics, enabling the cerebellum to adapt for the complex dynamics of multi-joint movements [92]. Cerebellar patients have difficulties in adjusting for the interaction torques occurring during fast reaches [12]. It has been repeatedly observed that during fast goal-directed movements cerebellar patients are unable to produce normal torque profiles. In particular, they show abnormal profiles in shoulder muscle torques varying inappropriately with the dynamic interaction torques occurring at the elbow joint. Magnitudes of dynamic interaction forces are scaled to the square of movement speed, an observation which might explain the worsening of dysmetria at higher velocities [53]. Inverse dynamic models allow for parsing the net forces acting at a joint into force components originating from muscular activation (MUS), external forces (EXT) including gravity, and dynamic inertial and interaction forces (DYN) [53]. The net torque (NET) is the sum of all positive and negative torque components:



In theory, dynamic interaction forces are the most critical component amongst dynamic movement variables during a coordination task or a multi-joint task. Dynamic interaction forces have to be precisely computed by the CNS. Since muscles are the end effectors, the selection of muscle activation patterns is a key step. Bernstein was the first to suggest that muscle activation is selected to compensate for physical consequences of motion [93]. Actually, the nervous system takes into account the fact that external forces and interaction forces may *support *or *antagonize *motion.

Given the numerous motor tasks and the huge number of interactions with the environment, it is widely accepted that the central nervous system must adapt quickly to the context [86]. In order to process all the contextual informations, it has been hypothesized that multiple controllers are in charge of a context or a small sets of contexts [72]. Indeed, a unique controller would demand an enormous complexity and would need to adapt each time to a new context, a potential source of errors [86]. This hypothesis takes into account the need to select the correct controller in a given circumstance [86]. To master this task, multiple paired forward-inverse models would be required.

#### Cerebellum and the adaptation of the magnitude of muscle responses to inertia or damping

Cerebellum tunes the intensity of the activities of numerous antagonist and synergist muscles used automatically in normal movements. It coordinates their timing, duration and amplitudes of activity [25]. A "tonic reinforcer" function seems suited for the interactions between the cerebellum and vestibular nuclei, reticular nuclei and motor cortex [25].

Fast single-joint monodirectional movements have been studied to extract specific patterns of muscle discharges in cerebellar patients. These movements are normally controlled by a triphasic pattern of EMG activity: a first burst in the agonist muscle (providing the launching torque) is followed by a second burst in the antagonist muscle (providing the braking torque), followed by a second burst in the agonist muscle (to bring the limb accurately to the target) [94,95]. Several deficits have been discovered in cerebellar patients (Figure 11): (a) a delayed onset latency of the antagonist EMG activity, (b) a slower rate of rise in the agonist/antagonist EMG activities, (c) an inability to tune the intensity of agonist/antagonist EMG activities when the inertia of the limb is increased [96,97].

**Figure 11:**
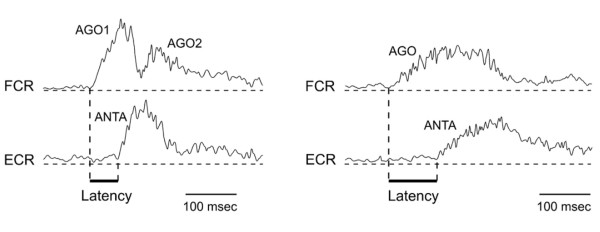
**Triphasic pattern of electromyographic (EMG) activities in a control subject (left) and in a cerebellar patient exhibiting hypermetria (right)**. In the control subject, the first agonist burst (AGO1) is followed by a burst in the antagonist muscle (ANTA), followed by a second burst in the agonist muscle (AGO2). In the cerebellar patient, three EMG deficits are observed: the rate of rise of EMG activities is depressed, the onset latency of the antagonist EMG activity is delayed and the 2 agonist bursts are not demarcated. FCR: flexor carpi radialis; ECR: extensor carpi radialis. EMG traces are full-wave rectified and averaged (n = 10 movements).

Recently, deficits in reversal movements have been found in ataxic patients. Reversal movements refer to movement towards a fixed target immediately followed by a return to the initial position. Reversal movements are balanced in shape, and the agonist EMG activity is composed of 2 bursts which are clearly separated [98]. During a fast voluntary movement, muscle damping, a non linear resistance to movement which depends on velocity, is typically asymmetrical, meaning that it predominates in the direction of muscle shortening [99]. For hand kinematics in the physiological range of motion, the damping compensation signal (aiming at compensating the asymmetry of the damping parameter) is a crucial element for kinetic encoding by the motor cortex [100]. The structures in the CNS regulating the damping compensation signal have not been identified so far, mainly due to technical constraints related to methods of investigations. The elucidation of the contribution of the cerebellar pathways in the damping compensation signal has remained so far elusive. In patients exhibiting a mild form of cerebellar ataxia, fast single-joint movements in one direction may be accurate. Thanks to the use of the haptic technology, it has been observed that these patients are unable to adapt to mechanical damping (addition of viscous forces) during the return to the initial position (second phase of the movement) [Figure 12]. The deficit is not dependent upon the initial direction of movement. In complex movements, the motor plan consists of *a superimposition *of elemental defined components [99,101]. In reversal movements, these elemental components need (1) to be selected and (2) to be superimposed sequentially. This highlights the fact that a given muscle can exhibit a normal behaviour facing mechanical damping during the first part of a motor sequence, but is not able to adapt appropriately for the next part. One implication is that current rehabilitation strategies in patients with cerebellar disorders should take into account the differences in the motor strategies underlying pointing movements and reversal movements in cerebellar disorders.

**Figure 12:**
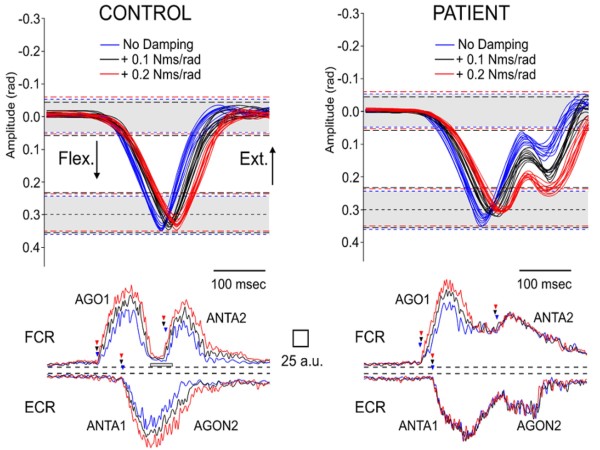
**Inability to adapt to damping in cerebellar hypometria during fast reverse movements**. Movement (top panels) and the associated EMG bursts in a control subject (left panels) and in an ataxic patient (right panels) for an aimed target of 0.3 rad are illustrated. Top panels: superimposition of fast reversal movements performed without damping (blue), with addition of 0.1 Nms/rad (black) or 0.2 Nms/rad (red). EMG bursts in the flexor carpi radialis (FCR) and the extensor carpi radialis (ECR) are calibrated with a reference to a maximal isotonic contraction (MIC) from 0 to 6 Nm (a.u.: arbitrary units). In each position panel, grey areas correspond to the 99% confidence interval of control values of movement amplitudes in the basal mechanical state (no addition of damping); dotted lines in black and red delineate the 99% confidence interval of control values during addition of 0.1 Nms/rad and 0.2 Nms/rad, respectively. In the patient, the first phase of movement (from the starting position to the target of 0.3 rad) remains accurate but the second phase (from the target of 0.3 rad to the return to the initial position) is hypometric. The hypometria is increased with addition of damping. Arrowheads located near the EMG traces indicate the onset of EMG bursts (blue: no damping, black: addition of 0.1 Nms/rad, red: addition of 0.2 Nms/rad). AGO1, AGO2 and ANTA1 correspond to the first burst in the FCR, the second burst in the FCR and the antagonist burst in the ECR, respectively. Arrowheads near AGO1, ANTA1, AGON2 and ANTA2 correspond to the onset of the first burst in the FCR, the first phase of the burst in the ECR, the second phase of the burst in the ECR, and the second burst in the FCR, respectively. AGO1 and ANTA2 are well demarcated in bottom left panel, unlike in the right bottom panel. Flex.: direction of flexion of the wrist; Ext.: wrist extension.

There are also experimental evidence that the cerebellum modulates the *gain *of reflexes in human. One example is that long-latency EMG responses (LLR) are abnormal in cerebellar patients. Typically, the first component M1 (of spinal origin) is spared in terms of latency/amplitude, whereas the magnitude of the M3 response (long-latency transcortical response) is increased [102,103]. This is illustrated in Figure 13. The phenomenon is particularly marked when lesions involve primarily the cerebellar cortex, suggesting a loss of inhibition from Purkinje cells leading to an overactivity of cerebellar nuclei. These data confirm a contribution of the cerebellum in the tuning of the magnitudes of muscle discharges.

**Figure 13:**
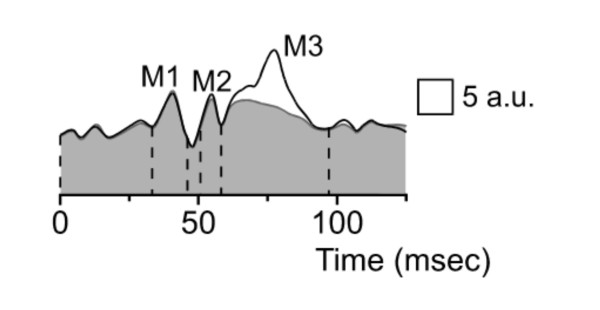
**Long latency electromyographic (EMG) responses to stretches of the first dorsal interosseous muscle in a cerebellar patient (black line) and in a control subject (grey line)**. Latencies of averaged rectified EMG responses are normal, but the M3 response is increased in the cerebellar patient. Surface EMG rectified and averaged 200 times. Responses are calibrated in arbitrary units (a.u.).

### Cerebellum as a movement timer

Another influential theory is that the cerebellum acts as a movement timer and is the main *site of temporal representation of action*, thanks to numerous interactions between the cerebellum and the inferior olive. Oscillations of inferior olive cells have been suggested to endow the system with the capacity to create complex temporal patterns, which might be applied for fine tuning of motor output and motor adjustments. Experiments showing that cerebellar lesions impair timing of motor acts are convincing [75,104,105]. Patients with lateral cerebellar lesions have difficulties in perceiving differences in intervals between tone pairs in the range of 0.5 sec, suggesting the presence of a general clock *not only for motion, but also for perception *[106]. Although apparently simple, the rhythmic synchronization between a timed sensory stimulus and a motor response step requires a highly complex signal processing procedure for the brain [107]. The production of a motor response time locked to a rhythmic stimulus implies an extraction of the timing information present in the sensory stimulus. Subsequently, this information has to be implemented to make predictions. Nevertheless, it is now clear that the cerebellum is not the sole site processing timing parameters in the brain [107]. The cerebellum, basal ganglia and frontal cortex interact strongly to pull out timing information and to funnel it in 'operative' centres. Cerebellar circuitry might work as a global support system in sensory acquisition and processing of timing procedures, facilitating the efficiency of brain networks [107]. The cerebellum could be seen as a sort of regulating clock.

### Cerebellum and sensori-motor learning

Damage to the cerebellum causes the inability to learn new complex movements [25]. Thanks to its high degree of adaptation in its operational mechanisms, the cerebellum contributes to various aspects of motor and non-motor learning. Motor learning can be defined as modifications in motor performance with practice, as an increase in the repertoire of motor behaviour or as a new behaviour maintained over a given period of time. In agreement with the theory of error signals, an increase in complex spikes firing rates during the adaptation phase to a novel load in a wrist-holding task has been demonstrated [108]. Once the task was learned, complex spike firing returned to baseline. According to Kitazawa and colleagues, complex spikes occurring early during a reaching task assists in encoding the absolute direction and destination of the arm, computing the relative endpoint errors of the reach [109].

There is strong evidence that eyeblink conditioning is dependent on the integrity of cerebellar networks. Findings in human are in good agreement with findings in animal studies [110]. Small lesions in the interpositus nucleus induce a permanent loss of conditioned responses. Several species have been used and several models of the basic neural circuits required for the acquisition and performance of classical eyeblink conditioning have been discussed [111]. An intermediate cerebellum-related network superimposed on the brainstem circuits regulating the inborn unconditioned eyeblink response has been proposed. Neural plasticity develops both in the cerebellar cortex and cerebellar nuclei following training [112,113]. Recent experimental observations are providing the first evidence that the memory trace of motor learning may shift trans-synaptically for consolidation to long-term memory [114]. Neuroanatomical correlates of learning have been studied in human. The majority of lesion studies have investigated conditioned response acquisition. The group of Timmann et al. has shown that the superior cerebellar artery supplies critical zones for eyeblink conditioning in human [110]. Cerebellar circuits are also involved in the timing and extinction of conditioned eyeblink responses. It should be mentioned here that regarding the vestibulo-ocular reflex (VOR) learning, experiments suggest that short-term learning is maintained by the cerebellum, while long-term learning can continue also when the cerebellum is removed.

### Other theories of cerebellar function

Coupling between the cerebellum and contralateral thalamic nuclei/primary motor cortex is well established [115]. Coherent oscillations between the sensory cortex and the cerebellar cortex have been reported. Activity in cerebellofugal fibers is triggering oscillations in thalamic nuclei and motor cortex [116]. The modulation of these oscillations in terms of frequency and synchronicity might be an important feature of the cerebello-cerebral loops. Moreover, another and complementary role for the cerebellum could be the tuning of the sensorimotor coupling of neural activities in a particular condition combining reflex and voluntary movement [117]. This theory is based on the fact that the relation between sensory signals guiding motion and the movement itself depends on the 'context', which takes into consideration the relative position of the limb segments, the position of the body in the gravitational field and the external forces interacting with the movement. This is also taken into account in the hypothesis of the *sensorimotor coordinate transformer*, according which the main function of the cerebellum is to mathematically transform signals from sensory to motor coordinates [118]. Cerebellar operations would be represented by a matrix of gains, leading to a prediction function.

The theory of the *wave-variable processor *attempts to explain how the cerebellum deals with the issue of feedback motor control in the presence of signal transmission delays [76]. The central premise of the wave-variable processor theory is that the interaction between the intermediate cerebellum and the spinal cord represent a wave-variable-based communication. This is based upon the teleoperation theory of Niemeyer and Slotine (1991) [119]. According to this theory, the cerebellum contributes to a servo-motor mechanism. The "servo hypothesis" was originally proposed by Merton [120]. Wave variables are linear combinations of command/feedback signals that can exchanged between a master unit and a slave unit to obtain a stable control whatever the transmission delay. The structure is consistent with the numerous combinations of inputs, such as force feedback signals and corollary discharges from internal pathways, ascending from the spinal cord via the spinocerebellar tracts. The wave-variable processing would allow the motor system to work without complex internal models. Simulations of rapid elbow movements have confirmed that the model mimics monkey's performance [76]. In addition, reduction of the cerebellar output induced a large oscillation reminiscent of cerebellar tremor. Interestingly, simulated signals of the interpositus nucleus matched the real signals recorded in monkey. This model can take into account the multiple reverberating loops existing between the cerebellum and brainstem nuclei (such as the cerebello-reticulo-cerebellar loops). However, due to its linearity, this model cannot be applied to the non-linear dynamics of a two-joint arm.

### What did we learn from studies including patients with cerebellar disorders?

Theories and models have been tested in cerebellar disorders encountered in the clinic, such as cerebellar stroke (acute focal lesion involving afferences and/or efferences) or the various forms of sporadic/inherited cerebellar degeneration (progressive loss of neurons in the cerebellum, especially in the cerebellar cortex). As mentioned in section III, the observation of the deficits in patients with cerebellar disorders argues in favour of a forward internal model [121]. Impaired adaptation in anticipatory responses is observed during various experimental paradigms [122,123]. For instance, it has been shown that anticipatory postural adjustments are under cerebellar supervision [82,122]. Horak and Diener have assessed the adjustments in torque responses during standing postural perturbations [122]. Healthy subjects are able to scale the anticipatory postural responses when perturbations are presented in a predictable order, unlike cerebellar patients. This suggests that the cerebellum plays an integral role in using predictive feedforward control to adapt postural responses [81]. Similar deficits have been reported in locomotor-like adaptation tasks [124]. Recent studies with a splitbelt treadmill (one leg is forced to move faster than the other) have demonstrated that the adaptation process includes both a reactive and a predictive component [125]. Reactive adaptations arise and disappear quickly, respectively after the perturbation and upon removal of the splitbelt condition [81]. Regarding the predictive adaptations, they become apparent after several strides and display after-effects suggesting that important informations related to the body-environment interactions have been stored. Whereas reactive adaptations are spared in case of cerebellar lesion, predictive adaptations are impaired [121]. In human, lesions of the dentate nucleus or lesions of the cerebellar cortex result in an uncoupling of grip force-load force during a lifting and holding task with objects of different weights [126]. By contrast, lesions in the territory of the posterior inferior region of the cerebellum do not cause any overshoot in grip force nor a lack of coordination between grip and load force profiles. The progressive and general loss of function encountered in hereditary spinocerebellar ataxias is also associated with impaired force adaptations during goal-directed arm movements [88]. The failure to generalize learning to untrained regions in the workspace suggests that a chronic and progressive loss of cerebellar circuits prevents the formation of the internal representation of limb dynamics. These findings have direct implications for daily rehabilitation of cerebellar dysmetria, but these are currently underestimated.

### Wearable devices and unobtrusive sensors

There are been limitations in the past to assess the mechanisms of dysmetria due to technical constraints. Wearable devices, unobtrusive sensors and body area networks, as well as new techniques of assessments such as haptic devices are emerging tools which will probably modify our understanding and our methods of clinical/laboratory evaluation of cerebellar dysmetria in the near future. Exoskeletons are a typical example of wearable devices with many potential applications in the field of motion research and therapy. For instance, they can be used to assess the effects of mechanical perturbations on cerebellar deficits such as cerebellar tremor [127]. They also can be used to evaluate their potential usefulness in restoring the metrics of motion in case of limb dysmetria. Moreover, these devices open new perspectives to assess the various theories of cerebellar functions in a clinical environment, especially by modifying the inertia or damping of individual segments in a given limb. Studies of eye-head-limbs coordination will benefit from technological developments in the coming years. Another example is the very recent application of brain-computer interfaces (BCI) in this area of research. Unobtrusive sensors are also becoming popular in functional imaging studies, where they can bring critical informations during data acquisition.

## Conclusion

**Figure 14:**
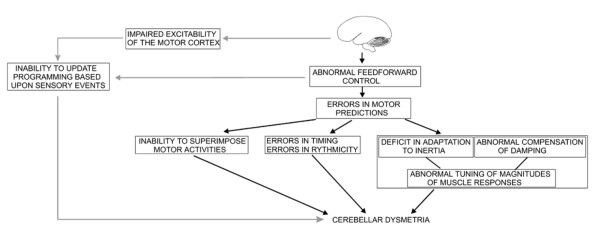
**Overview of the mechanisms of human cerebellar dysmetria**.

**Figure 15:**
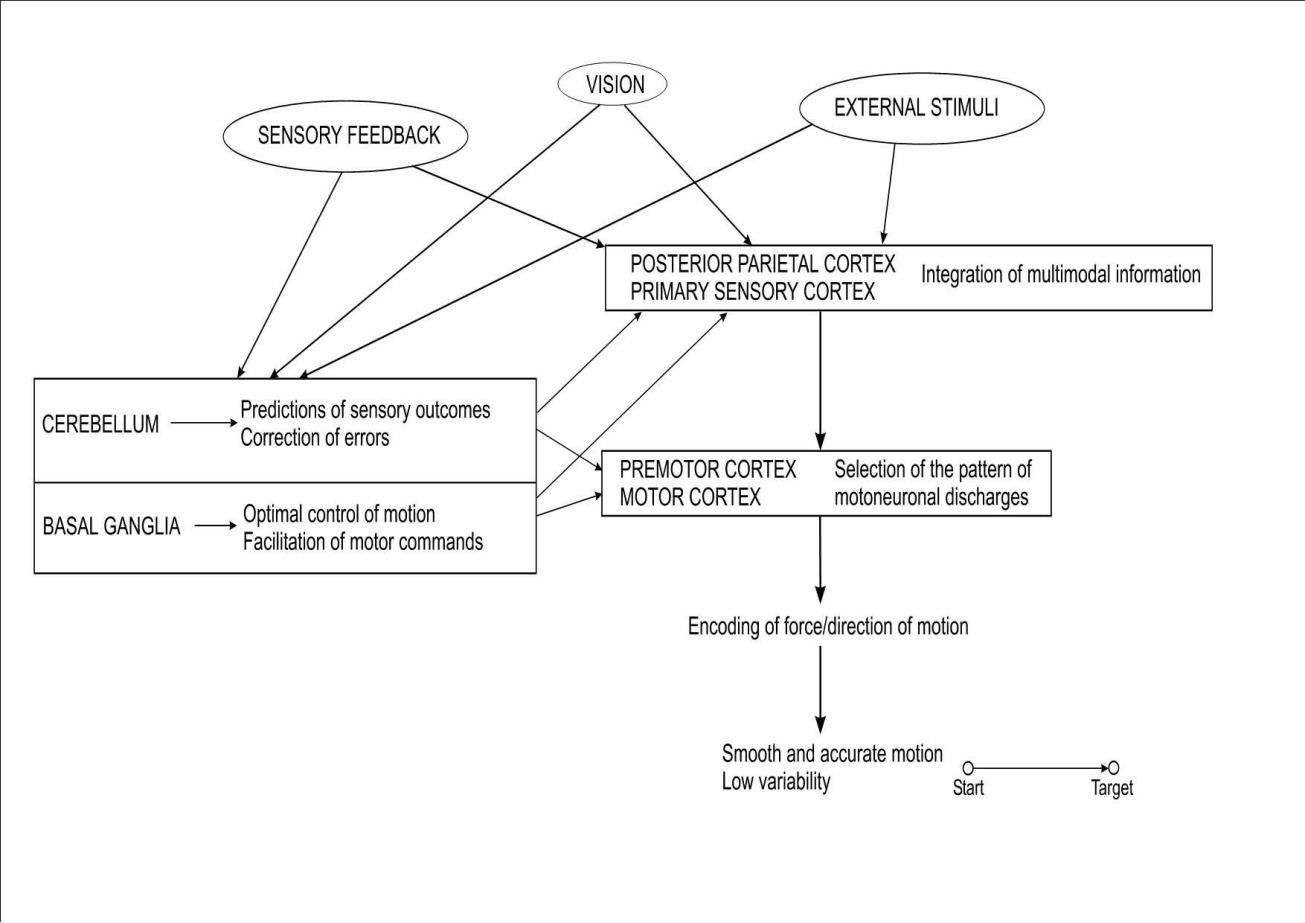
**Overview of the motor control strategy for limb movements**. Cerebellum builds internal models and corrects motor commands, comparable to a system identification function. Basal ganglia ensures an optimal control of motion, facilitating motor commands. The parietal cortex integrates proprioceptive and visual outcomes, as well as sensory feedback, playing a role of state estimator. Premotor cortex and motor cortex transforms predictions into sets of motoneuronal discharges, encoding for force and direction of movement.

**Figure 16:**
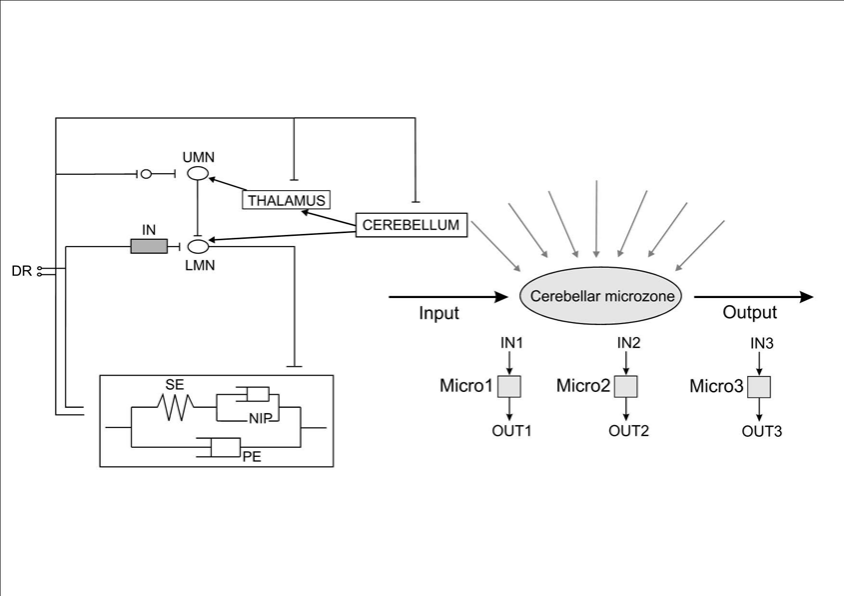
**Representation of the sites of action of the cerebellum**. Hill's muscle model and an operational model of the cerebellar circuitry are illustrated. Central and peripheral loops in the central nervous system are shown, with upper motoneuron (UMN)/lower motoneuron (LMN). IN indicates the pool of interneurons in the spinal cord. Cerebellar influences on spinal motoneurons are mainly indirect. DR corresponds to the dorsal root ganglia. The rectangle in the bottom represents Hill's muscle model (SE: series elastic component; NIP: neural input processor in parallel with a viscous component PE). Operational model of the cerebellar circuits: given inputs (IN) to a microzone (Micro) elicit an output (OUT) which depends on the whole complex of afferent information impinging upon the microzone. Adapted from Manzoni, 2007.

We have provided an overview of the current theories underlying the roles of the cerebellum in motor control and the mechanisms of cerebellar dysmetria. From the anatomical point of view, the cerebellum is very well positioned *to process multi-modal sensory information*. On the basis of available experimental data, it can be proposed that cerebellar dysmetria mainly results from a deficit in the predictive feedforward control (Figure 14). Predictions and subsequent updates based on sensory events would be possible thanks to the numerous projections received by the cerebellum, the huge computing capabilities of the cerebellar circuitry, and the pertinent interaction of mossy and climbing fibres [86]. Cerebellar cortex, especially Purkinje neurons, plays a key-role in coding the kinematic features of movement. Through the cerebello-thalamo-cortical channel, inputs can modulate the efficacy of the interconnections among cortical neurons, adjusting the circuitry of the motor cortex in various contexts and implementing predictions in the sensorimotor system. As a result of a cerebellar lesion, patients have a disorganized timing implementation in motor tasks and exhibit difficulties in tuning the magnitudes of motor responses. The generation of inappropriate muscle torques may result from the errors in the prediction of the mechanical consequences of movements of one limb segment on adjacent joints [53]. Errors in predicting compensation torques may cause the abnormal metrics of motion (dysmetria). Both the defect in feedforward control and the abnormal excitability of the motor cortex result in an inability for the motor system to update motor programming based upon sensory events. Cerebellar circuitry would cooperate with basal ganglia to generate smooth movements, despite state changes and time delays (Figure 15). A function of the cerebellum is system identification. The cerebellum would construct internal models with the aim of predicting sensory outcome of motor commands and correct these commands via internal feedback [128]. Cerebellum acts both at the upper motor neuron and lower motor neuron level to tune muscle discharges and modulate the muscle reactivity to environmental changes. Figure 16 summarizes the interactions between the cerebellum and the motor system using the component-based Hill muscle model, which is commonly used to predict muscle forces and which represents the active and passive properties of the musculo-tendinous unit [129]. The model includes a series elastic component and a neural input processor in parallel with a viscous component. The figure also shows an operational model for the cerebellar circuits.

The current theories of cerebellar functions can be understood as complementary rather than mutually exclusive. Some of them share commonalities. For goal-directed tasks, predictive control is essential for fast execution, but predictions are also important for slow motion, due to the increased reliance on time-delayed feedback signals [130]. The combination of both forward and inverse models results in computational advantages for motor learning and control. The context of the experiments, the biomechanical features of the effectors being considered (eyes, limbs,...), the motor task (reaching task, grasping, postural task, gait,...), the way data have been collected, and the clinico-radiological aspects (in case of studies with patients) should all be taken into account and integrated when attempting to extract the conceptual bases underlying cerebellar dysmetria. Quantitative lesion approach and theoretical motor control provide complementary informations.

## Competing interests

The author declares that they have no competing interests.
